# Conference Report: SIXTH INTERNATIONAL CONFERENCE ON THE APPLICATION OF STANDARDS FOR OPEN SYSTEMS, Gaithersburg, MD, October 2–4, 1990

**DOI:** 10.6028/jres.096.051

**Published:** 1991

**Authors:** Shirley M. Radack

**Affiliations:** Computer Systems Laboratory, National Institute of Standards and Technology, Gaithersburg, MD 20899

## 1. Introduction

Worldwide interest in advancing open computing systems was highlighted at the Sixth International Conference on the Application of Standards for Open Systems. Held in the United States for the first time, the conference was co-sponsored by the IEEE Computer Society, the Institute of Electrical and Electronics Engineers, and the National Institute of Standards and Technology. An exhibit of technical and educational products related to open systems was organized by the Corporation for Open Systems.

Chaired by Kevin Mills, Chief of the Systems and Network Architecture Division at the Computer Systems Laboratory, the conference featured papers and discussion on key issues affecting the implementation of open systems including policy development, international collaboration, trade issues, implementation, conformance, and security.

The conference committee included Allan Maclean of Australia, William McCrum of Canada, Michel Audoux of the European Community, Bernard Gondran and Claude Mahy of France, Hideaki Okino of Japan, George Sidey of the United Kingdom, James H. Burrows of the United States, and Wolfram Berger and Heinrich Wortmann of West Germany.

## 2. Requirements for Open Systems

Requirements for open systems are being driven by users who want to move away from proprietary systems to standard interfaces and interoperable software, hardware, and communications products that are developed by different vendors. No single vendor can supply systems to meet the diversity of many user requirements, or respond to enterprise-wide needs for common application architectures, communications, and networks. Users need flexible and modular systems that can be acquired and added to with equipment supplied by a variety of vendors in an open competitive market, and that can support the portability of software applications.

## 3. Opening the Conference

In welcoming the conference participants, James H. Burrows, Director of the Computer Systems Laboratory, said that “the cooperative efforts of users, governments and industry have taken us down the road toward open systems,” and that efforts must be maintained to continue to build on these achievements and to augment open systems standards and applications. Mr. Burrows cited the need for standards to protect the confidentiality, integrity, and availability of information transmitted in global networks for electronic mail, business data interchange and other strategic business functions.

Robert White, Under Secretary of Commerce for Technology, delivered the keynote address. In his talk. Dr. White said that new alliances will be needed to unify and to present user requirements for open systems to industry and to standards organizations. He announced that the U.S. Government was forming the Federal Open System Users Council comprising senior-level government executives. The council will develop common architectural frameworks for open systems to meet government requirements for interconnection and interoperation of systems from different vendors and for the portabilify of software. The Council will work with users and vendors to promote better understanding of common requirements.

Dr. White said that open systems, based on standards, will integrate software and hardware components, and provide standard interfaces to application programs and to the user. But, these systems must be based on industry-wide standards, be commercially available, and be capable of being extended or modified. A consensus based process for decisions regarding definitions, specifications and other issues must be available.

Calling for an integrated world market for open systems. Dr. White said that these markets would allow for competition based upon price, performance, and added value. Such competition helps buyers by lowering prices and improving quality. He also stated that the development of tests was essential to give users confidence that systems conform to standards and will interoperate. International cooperation among standards groups, technical organizations, and governments is needed to achieve mutual recognition of tests, testing methods, and test results. Such efforts will be effective in minimizing regional differences, reducing the need for multiple product testing cycles, and getting products to users more quickly and cheaply, he said.

## 4. Conference Papers

The proceedings of the Sixth International Conference on the Application of Standards for Open Systems were published by the IEEE Computer Society Press, 10662 Los Vaqueros Circle, P.O. Box 3014, Los Alamitos, CA 90720-1264. Most of the presentations referenced below are included in the proceedings.

**New Open Systems Interconnection (OSI) Policies**
  Johansson, B., OSI Strategies for Scandinavian Governments
    Statsliontoret
    Sweden
  Oldno, H., Japanese Activities for Promoting OSI
    Ministry of Trade and Industry
    Japan
  Ramakrisimam, S., OSI Policy in India
    Department of Electronics
    India
  Houser, W., Implementing GOSIP in VA
    Department of Veterans Affairs
    United States
**International Collaboration**
  Hartmann, U., Open Systems Standards: Status of International
    Harmonization and European Activities
    ZVEI (Siemens)
    Federal Republic of Germany
  Read, C, Global Harmony, The Delivery of Proof
    Digital Equipment Co., Ltd.
    United Kingdom
  Therrien, J., International Collaboration in the Public Sector
    Treasury Board Secretariat
    Canada
**Free Trade and Standards**
  Mills, K., Standards and Trade: What’s the Connection?
    National Institute of Standards and Technology
    United States
  Cameron, P., Impact of Trade Agreements on Standardization
    Canadian General Standards Board
    Canada
  Wilkinson, C, Economic Implications of Standardization:
    OECD
    GATT
    CEC, DGXIII
    Belgium
**Applications**
  Tunstall, J., Using OSI for the Exchange of Information between Financial Institutions
    Association for Payment Clearing Services
    United Kingdom
  Pinson, PH., Business Case for Open Systems
    Dupont
    United States
  Ochiai, T., Numura OSI: An Example of the OSI-Based
    Transaction Processing
    Nomurs Research Institute, Ltd.
    Japan
  Ono, K., The Applications OSI to ISDN Promotion in Japan
    The Telecommunication Technology Committee
    Japan
  Staudinger, W., Telematic Terminals for ISDN: OSI As A Market Drive or a Conflict with User Needs?
    Deutsche Bundespost TELEKOM
    Federal Republic of Germany
  Corrigan, M. L., The Integrated Federal Telecommunications
    System (IFTS)
    General Services Administration
    United States
  Calder, C, The Emergence of Information
    Networks The Automobile Association
    United Kingdom
  Virol, L., Vans and OSI Promotion
    Ministere PTE
    France
  Becker, I. B., Electronic Data Interchange by UN/EDIFACF
    Standards
    VDMA (IBM)
    Federal Republic of Germany
  Dreyfous, E., The Integration of Trade and Industry EDI
    Requirements in International Telecommunications
    EDIFRANCE
    France
**Applications Portability**
  Hankinson, A., Open System Standards for Application Portability
    National Institute of Standards and Technology
    United States
  Saito, N., Japanese Standardization Activities for the Interfaces for Application Portability (IAP)
    Keio University
    Japan
  Griffiths, P., Technological Change, Distributed Processing, and Applications Portability
    The Instruction Set, Hoskyns Open System Division
    United Kingdom
**Conformance and Interoperability Assurance**
  Mulvenna, G., The OSINET Testing and Registration Service
    National Institute of Standards and Technology
    United States
  Nilsson, S., ETIC–The European System for FT Testing and Certification
    ECITC
    Sweden
  d’Oultremont, P., COS/SPAG/POSI Open Integrated Tool Set SPAG
    Belgium
  Asano, S., Conformance Testing, Certification and Interoperability Assurance in Japan
    National Center for Science Information System
    Japan
  Favreau, J., The U.S. GOSIP Testing Program
    National Institute of Standards and Technology
    United States
  Davis, W., Canadian Open Systems Testing
    Canadian Open Systems Testing Corporation
    Canada
  Corsi, N., Conformance Testing Services in Europe
    Open Systems Testing Consortium
    Italy
**Security**
  Troye, A., Legal Problems of Electronic Documents
    CEC, DGXIII
    Belgium
  Kowalski, B., Security Protocols for Open Communications
    Deutsche Bundespost TELEKOM
    Federal Republic of Germany
  Wood, J., European Harmonised IT Security Evaluation Criteria
    Department of Trade and Industry
    United Kingdom
**The Future**
  Pouzin, L, Ten Years of OSI, Maturity of Infancy
    THESEUS
    France
  Kahn, R., Open Systems in Future Technology
    National Research Initiatives
    United States

